# The Cross-Sectional Evaluation of the Use of Artemisinin-Based Combination Therapy for Treatment of Malaria Infection at a Tertiary Hospital in Nigeria

**DOI:** 10.1155/2018/2025858

**Published:** 2018-05-16

**Authors:** Roland Nnaemeka Okoro, Muslim Olakunle Jamiu

**Affiliations:** ^1^Department of Clinical Pharmacy and Pharmacy Administration, Faculty of Pharmacy, University of Maiduguri, Maiduguri, Nigeria; ^2^Department of Clinical Pharmacy and Pharmacy Practice, Faculty of Pharmaceutical Sciences, University of Ilorin, Ilorin, Nigeria

## Abstract

In 2005, Nigeria changed its antimalarial drug policy to Artemisinin-based Combination Therapies (ACTs) for the treatment of malaria infection, and it is imperative for prescribers to strictly comply with this guideline to harmonize malaria management practices within the country. This study aims to evaluate prescribers' adherence with the National Antimalarial Treatment Guideline (NATG) in the treatment of malaria infections and to describe the determinants of antimalarial drugs coprescription with antibiotics at a tertiary hospital in Nigeria. A cross-sectional, retrospective study of antimalarial drug prescriptions of one-year period of 2013 was conducted. A simple method for assessing the quality of drug prescribing (DU90%) was adopted. Logistic regression was used to predict antimalarial drugs coprescription with antibiotics. Overall, 95.8% of the total prescriptions contained ACTs, out of which 80.8% were Artemether/Lumefantrine. However, adherence to NATG was 88.2% with an adjusted value of 100.0%. Age was the only predictor for antimalarial drugs coprescription with antibiotics. This study showed high concordance with NATG at the studied hospital. Age less than 5 years is a significant risk factor for antimalarial drugs coprescription with antibiotics.

## 1. Introduction

Malaria remains the most common public health problem in Nigeria where it accounts for more cases and deaths than any other country in the world. Malaria is a risk for majority of Nigeria's population with an estimated 100 million malaria cases with over 300,000 deaths per year in Nigeria [1]. Children under 5 years of age are the most vulnerable group and account for significant malaria deaths [2]. The aim of proper malaria diagnosis and treatment is to cut the disease burden, death, and socioeconomic losses. The use of national guidelines for malaria diagnosis and treatment is paramount to achieving this goal. The antimalarial drug policy was formulated to promote rational use of antimalarial drugs in order to reduce the development of drug resistance.

Following a period of continuous increase in resistance of* Plasmodium falciparum* against the commonly used antimalarial drugs such as chloroquine, and Sulphadoxine-Pyrimethamine (SP), the new artemisinin-based combination therapy (ACT) was introduced in 2005 with Artemether-Lumefantrine (AL) as first-line treatment for uncomplicated malaria and Artesunate + Amodiaquine (copackaged) as alternative [3, 4]. However, the meta-analysis conducted in 2014 reported that the prevalence of use of ACT in the public sector in Nigeria was 76% [5].

To meet the goal of universal access to right interventions for all populations at risk of malaria, it is required that the proper clinical investigation is conducted prior to treatment with effective antimalarial drugs [4]. However, one of the main obstacles to health care access is the direct out-of-pocket payment form of health care financing which occurs in most sub-Saharan African countries [6]. In a bid to promote access to quality health care services, Nigerian government introduced National Health Insurance Scheme (NHIS) in 2005. Evidence shows that the National Health Insurance policy has led to an increase in utilization of medicines [7]. In spite of this, as at 2016 the scheme has covered only about 4 percent of total Nigerian population [8].

Appropriate treatment of malaria and the correct use of antimalarial drugs are needed in order to achieve Nigerian's goal of preelimination and reducing malaria related death to zero by 2020 [9]. Drug use studies are carried out to find out associated problems with drug use since adherence of health providers and patients to recommendations of a treatment policy is key to the overall success of such guidelines. Drug use studies could unravel drug use problems and give feedback to the prescriber to create awareness on rational use of drugs [10]. As such, antimalarial drug use studies are important in an effort to redress the impact of malaria as it will make sure that care providers in the health insurance scheme are working in concert with national strategy.

In Nigeria today, there is paucity of data on the implementation of this new antimalarial policy in the NHIS. An NHIS antimalarial prescription audit has been done in the South Western part of the country [11]. To our knowledge, no antimalarial drug use study has been conducted in NHIS in the South Eastern Nigeria, hence the need for this study. It is necessary to survey antimalarial drug use among insured patients at a tertiary hospital in this part of the country to offer insights into provider's antimalarial drugs prescribing practices and to gather baseline information that could serve as a basis for designing appropriate interventions and policies to improve antimalarial drugs use within the scheme. The aims of this study were to evaluate prescribers' adherence with the National Antimalarial Treatment Guideline in the treatment of malaria infections and to assess the determinants of antimalarial drugs coprescription with antibiotics in the NHIS at tertiary hospital in Nigeria.

## 2. Method

### 2.1. Study Site and Design

This was a descriptive, cross-sectional, retrospective study of prescriptions purposively carried out among NHIS outpatients at the University of Nigeria Teaching Hospital (UNTH), Enugu. NHIS outpatients are seen by medical doctors from various specialties. These patients access their NHIS approved drugs with a 10% copayment only from the NHIS outpatients' pharmacy unit. UNTH is a tertiary health care facility of about 500-bed capacity with staff made up of professionals and nonprofessionals. It serves as the teaching hospital for the faculty of medicine of the University of Nigeria and is a participating Health Care Provider (HCP) on the insurance scheme.

### 2.2. Sampling

This comprised all NHIS outpatients' prescriptions that contained at least one antimalarial drug filled from January to December 2013. Prescriptions from antenatal clinic were excluded due to the use of SP for Intermittent Preventive Therapy (IPT) in pregnancy.

Large sample size which exceeded the minimum of 100 suggested by WHO was employed in order to enhance the reliability of the results since only one health facility was used for the study [10]. Systematic sampling was used to select the prescriptions. Based on the total number of 6667 prescriptions containing at least one antimalarial drug after excluding those from antenatal clinic, a sampling interval of 20 was calculated and simple balloting was used for the first pick. At the end, a total number of three hundred and thirty-three (333) prescriptions containing at least one antimalarial drug were selected for the study.

### 2.3. Data Collection

The modified World Health Organization (WHO) prescribing indicator form was used to extract the following data: age; sex; month of prescription; the names of antimalarial drugs prescribed; number of drugs prescribed; number of drugs dispensed; number of drugs prescribed from Essential Drug List (EDL) [12]; number of drugs prescribed by generic name; encounter with an antibiotic prescribed; and encounter with an injection prescribed. These data were later entered into Microsoft Excel (Microsoft Corporation, 2007).

### 2.4. Data Management and Analysis

Abstracted information was later keyed into Statistical Package for Social Sciences (SPSS, version 21, Chicago, USA) coded for data analysis. Raw data were double-checked with soft data for consistency. The WHO drug use indicators investigated in this study were the average number of drugs per encounter, percentage encounter with an antibiotic, percentage encounter with an injection, number of drugs prescribed by generic name, and the number of drugs prescribed from NHIS EDL described elsewhere [10]. Drug use metric employed was number of prescriptions and drug use pattern was expressed in terms of Drug Utilization Ninety Percent (DU90%). The DU90% segment reflects the number of drugs that account for 90% of drug prescriptions. Adherence with the regimens listed in the National Antimalarial Treatment Guideline (NATG) was assessed [13]. Adherence was defined as number of prescribed regimens recommended from the NATG divided by number of regimens in the DU90% segment, expressed in percentage. Adjusted adherence represents the percentage of all regimens listed in the NATG in addition to other ACTs not listed in NATG. Logistic regression was used to model the predictors on the outcome variable (antimalarial drug coprescription with antibiotics). Statistical significance was set as a *p* value less than 0.05 (*p* < 0.05).

### 2.5. Ethics

Permission to conduct the study was sought from the hospital management and ethical clearance was obtained from the Research and Ethics Committee of the UNTH.

## 3. Results

### 3.1. Patients Baseline Demographic Characteristics

Demographic information of the study patients is presented in Table 1. In total, 333 prescriptions containing antimalarial drugs were reviewed. It was found that more than one-half (69.4%) of the prescriptions were written for female patients. About 54.0% of the patients were older than 18 years (Table 1).

### 3.2. Prescription Pattern of Antimalarial Drugs

Table 2 shows that, among the ACTs and nonartemisinin monotherapy, AL and Proguanil were the most prescribed antimalarial drugs, respectively. On evaluation of the artemisinin monotherapy prescribed, it was found that one-half (Artemether 16.7% and Arteether 33.3%) was given as injectables.

### 3.3. Drug Utilization Ninety Percent (DU90%) of Antimalarial Drug Regimen

**Figure 1:**
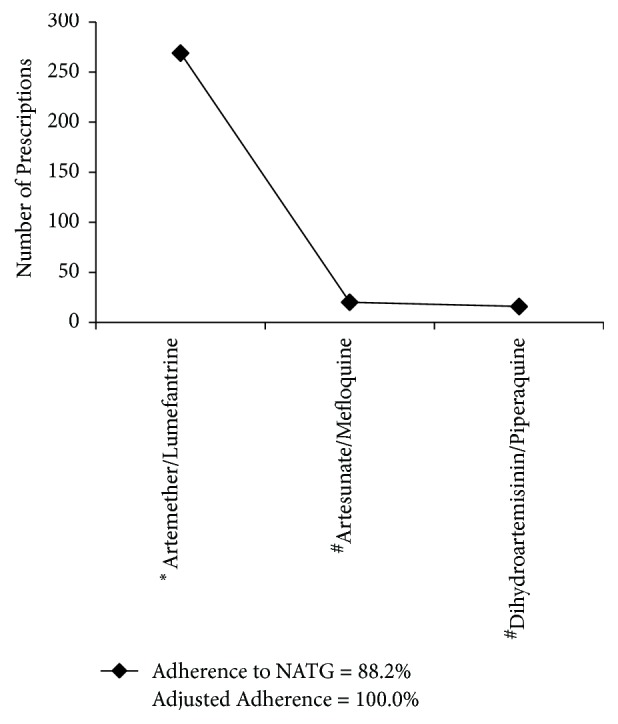
*Drug utilization ninety percent (DU90%) of antimalarials*. ^*∗*^Antimalarial regimen listed in the NATG. ^#^Antimalarial regimens that are not listed in the NATG, but can be referred to as “recommended” treatment as they contain an artemisinin derivative and partner drug. Adherence: Based on antimalarial regimens listed in the NATG. Adjusted Adherence: Based on all ACTs.

Figure 1 shows the antimalarial regimens within the drug utilization (DU90%) segment. Only three drugs appeared in the DU90% segment with a high adherence of 88.2% to NATG and adjusted adherence of 100.0%.

### 3.4. Prescribing Indicators

Table 3 shows the drug use indicators. The total number of drugs prescribed was 1,585 with an average number of drugs per prescription of 4.8. The maximum number of drugs prescribed per prescription was 11, representing 0.6% of all prescriptions. The median number of drugs per prescription was 5.

### 3.5. Determinants of Antimalarial Drugs Coprescription with Antibiotics

Table 4 summarizes the results of predictors for antimalarial drugs coprescription with antibiotics. Age was the only significant predictor for antimalarial drugs coprescription with antibiotics.

## 4. Discussion

This study revealed high prevalence of use of ACTs especially AL for the treatment of malaria infections among insured patients. AL is the antimalarial drug of choice for the treatment of uncomplicated malaria in Nigeria due to its demonstrated efficacy. The results of the drug efficacy trials carried out in the all the geopolitical regions of the country in 2004 found AL to be highly efficacious and thus suitable for use in the treatment of uncomplicated* P. falciparum *malaria in the country [3]. This finding is consistent with the findings of other Nigerian studies [14–17], but inconsistent with a previous NHIS study done in another part of the country [11]. Secondly, there was high concordance with NATG at the study setting which is commendable. Interestingly, only three antimalarial drug regimens made it to the DU90% segment. This result is suggestive of high quality prescribing of antimalarial drugs in the setting studied. It has been found that the use of larger number of choices of therapy results in a decreased rational clinical decision-making process [18]. Therefore, high quality prescribing is associated with the use of a relatively limited number of pharmaceutical products [19]. Besides high quality prescribing, our study recorded a higher adherence to NATG compared to a non-NHIS study that reported lower levels of 66.7% in secondary hospital and 38.5% with adjusted adherence of 69.2% in a tertiary hospital. Our finding demonstrates the usefulness of NHIS as a tool towards realizing the goal of eliminating malaria related morbidity and mortality in Nigeria by 2020.

Analysis of drug prescribing indicators revealed high average number of drugs prescribed per encounter which indicates occurrence of polypharmacy. This result is comparable with the values of 3.4, 3.8, and 4.1 got from earlier NHIS studies in tertiary hospitals in South Western, North Eastern, and North Western Nigeria [7, 20, 21]. A plausible reason for this polypharmacy in NHIS at the tertiary hospitals might be reasonable in some cases due to high prevalence of comorbidity, while unjustifiable ones could be attributed to patients' demand for drugs and the fact that NHIS is a subsidized scheme in which patients pay only 10% of the medication fees as copayment. The current study also revealed low generic prescribing which is congruent with other Nigerian studies done in the NHIS [7, 20, 21]. Low value was found in these studies despite the fact that Nigerian National Health Insurance Policy, published in 2005, prescribed that purchase and prescribing of drugs in the NHIS shall be by generic names only. The implications of low generic use are primarily the wastage of scarce health resources and a decrease in access to pharmaceuticals because of affordability barrier to patients if medicine out of stock occurs in the NHIS.

However, evaluation of encounters with antibiotics revealed a higher value of 37.2% as against the WHO reference range of 20.0%–26.8% [22]. Other NHIS studies have also reported values above WHO reference range [7, 20]. Concomitant prescription of antimalarial drugs and antibiotics occurs usually to take care of any potential coinfection implying that the prescriber is not too certain about the real diagnosis, or to prevent undetectable infection showing up [23]. Overuse of antibiotics leads to emergence of drug resistance microorganisms and increased side effects and cost of healthcare. Therefore, this finding serves as a clarion call for improved diagnosis of nonmalarial fever and to physicians to prescribe antibiotics with caution to cut these negative health outcomes.

The low number of drugs prescribed from the EDL was due to nonrevision of NHIS EDL 2005 edition for almost a decade. Implementation of this EDL after 8 years constrained the prescribers in the scheme, thereby forcing them to prescribe some drugs not listed in the NHIS EDL that were considered effective based on clinical judgment to meet the desired health outcomes for the patients. The concept of essential drugs incorporates the need to regularly delete obsolete medicines and add newer more effective ones to reflect new treatment options and changing therapeutic needs among others [24].

The low use of injections and high amount of medicine dispensed were commendable. Use of injections is associated with adverse events and increased cost. On the other hand, high number of medicines dispensed reported by this study shows that the hospital procured some medicines outside the NHIS EDL to decrease patients out-of-pocket expenses on medicines due to the restriction imposed by the outdated NHIS EDL.

Furthermore, this study assessed the factors associated with antimalarial drugs coprescription with antibiotics. Predictor found to be associated with the risk of being coprescribed antimalarial drugs with antibiotics was age. Children under five years of age were much more likely to be coprescribed antimalarial drugs and antibiotics than those aged 5 years and more. Similar findings were reported in South Eastern and South Western Nigeria [17, 25], Ghana [23], and India [26]. This might be due to paediatrics having high risk to suffer from recurrent infections of other systems such as the respiratory tract (cough) and gastrointestinal system (diarrhoea).

The limitations of this study include the retrospective collection of prescription data. This made it impossible for parasitological and patient clinical results to be collected. Therefore, use of drug could not be linked with the clinical decisions that informed the prescriptions. However, only prescription data was used to describe quality indicator index of drug prescribing: DU90%. Due to time lag between data collection and publishing of findings, the study findings may not reflect the current antimalarial drug prescribing practice of the setting studied. Lastly, the use of only one centre for the study restricts the generalization of the findings.

## 5. Conclusions

This study showed a higher use of artemisinin-based combination therapy and high concordance with National Antimalarial Treatment Guideline for the treatment of malaria infection. Based on this result, policy makers can consider addressing Universal Health Coverage (UHC) as health priority for Nigerian citizens as part of national malaria elimination strategy. Age less than 5 years is a significant risk factor for antimalarial drugs coprescription with antibiotics We recommend that prescribers at the study setting should conduct appropriate test to confirm bacterial infections before prescribing antibiotics especially for this vulnerable age group.

## Figures and Tables

**Table 1:** Demographic characteristics of patients prescribed antimalarial drugs (*N* = 333).

Variable	*n* (%)
*Gender*	
Female	231 (69.4)
Male	104 (30.6)
*Age group (years)*	
<5	21 (6.3)
5–11	24 (7.2)
12–18	11 (3.3)
>18	179 (53.8)
Not indicated	98 (29.4)
*Treatment period *	
First quarter	80 (24.0)
Second quarter	125 (37.5)
Third quarter	118 (35.4)
Fourth quarter	10 (3.0)

A quarter refers to one-fourth of a year (a period of 3 months).

**Table 2:** Prescription pattern of antimalarial drugs (*N* = 333).

Antimalarial drug regimens prescribed	*n* (%)
*Non-artemisinin monotherapies (NAMTs)*	*8 (2.4)*
Proguanil	5 (1.5)
Sulphadoxine-Pyrimethamine	2 (0.6)
Amodiaquine	1 (0.3)
*Artemisinin monotherapies (AMTs)*	*6 (1.8)*
Artesunate	2 (0.6)
Arteether	2 (0.6)
Artemether	1 (0.3)
Dihydroartemisinin	1 (0.3)
*Artemisinin combination therapies (ACTs)*	*319 (95.8)*
Artemether/Lumefantrine	269 (80.8)
Artesunate/Mefloquine	20 (6.0)
Dihydroartemisinin/Piperaquine	16 (4.8)
Artesunate/Amodiaquine	8 (2.4)
Artesunate/Piperaquine	4 (1.2)
Artemether/Lumefantrine + Sulphadoxine-Pyrimethamine	1 (0.3)
Artesunate/Sulphadoxine-Pyrimethamine + Proguanil	1 (0.3)

**Table 3:** Drug prescribing indicators.

Variable	Value
Average number of drugs prescribed per encounter (mean ± SD)	4.8 ± 1.8
Percentage encounter with an antibiotic (%)	37.2
Percentage encounter with an injection (%)	1.5
Percentage of drugs prescribed by generic name (%)	49.3
Percentage of drugs prescribed from EDL (%)	63.0
Percentage drugs dispensed (%)	91.8

**Table 4:** Predictor variables for antimalarial drug coprescription with antibiotics.

Variable	Adjusted odds ratio	95% CI	*p* value
*Age group (years)*			
<5 years	Reference		
5–11 years	0.16	0.04–0.61	0.007
12–18 years	0.20	0.04–1.03	0.055
>18 years	0.29	0.11–0.75	0.011
*Gender*			
Female	Reference		
Male	0.86	0.52–1.42	0.551
*Treatment period *			
First quarter	Reference		
Second quarter	0.87	0.48–1.58	0.650
Third quarter	0.94	0.51–1.72	0.837
Fourth quarter	1.72	0.44–6.76	0.441

## Supplementary Materials

Supplementary Materials Additional file (xls) containing antimalarial drug information and drug prescribing indicators data.
